# Music Listening and Stress in Daily Life—a Matter of Timing

**DOI:** 10.1007/s12529-017-9697-5

**Published:** 2017-11-21

**Authors:** Alexandra Linnemann, Mario Wenzel, Jennifer Grammes, Thomas Kubiak, Urs M. Nater

**Affiliations:** 1 Department of Psychology, University of Marburg, Marburg, Germany; 2 Health Psychology, Johannes Gutenberg University Mainz, Mainz, Germany; 3 Department of Psychology, University of Vienna, Liebiggasse 5, 1010 Vienna, Austria

**Keywords:** Ambulatory assessment, Duration, Health, Music listening, Stress, Temporal dynamics

## Abstract

**Purpose:**

Despite increasing evidence suggesting that music listening in daily life has stress-reducing effects, studies mostly rely on subjective, retrospective data on music listening. Thus, the temporal dynamics underlying the stress-reducing effect of music listening remain unclear. Therefore, we aimed to examine the temporal dynamics of the associations between stress and music listening by assessing subjective and objective data on music in daily life.

**Design:**

An exploratory Ambulatory Assessment study examining a total of 60 participants (37 women), aged 18 to 34 years (*M* = 22.4 years, SD = 3.5) was conducted.

**Methods:**

For 1 week, participants answered questions on music listening and stress six times per day via an electronic diary device, which additionally objectively sampled the exact time point of music listening and its duration.

**Results:**

Self-reports on mere music listening were associated with lower stress reports, whereas objectively assessed data was not. However, concerning duration of music listening, both subjective and objective data on music listening showed associations between a minimum of 20 min of music listening and lower stress reports. Concerning the latency, objective data on music listening revealed that the association between stress reports and music listening occurs in a time-delayed manner.

**Conclusions:**

Although the study design does not allow for causal inferences, substantial associations among subjectively and objectively assessed data on music listening were found to differentially affect the experience of stress after music listening. In particular, when focusing on the temporal dynamics, objectively assessed data allowed for a more fine-grained analysis. In consequence, subjectively and objectively reported data on music listening should be assessed jointly when investigating effects of music listening on health. Experimental research with rigorous methodological control is required in order to corroborate our findings in a laboratory setting.

## Music Listening and Stress in Daily Life—A Matter of Timing

Music listening in daily life may reduce stress [1]. Most of the evidence on this stress-reducing effect was gathered in quasi-experimental studies [2]. However, findings from these studies are quite heterogeneous, as they differ in terms of experimental design, music selection, and participants, making comparisons across studies difficult. Furthermore, in such quasi-experimental studies, participants are most often investigated only once, in one artificial setting (e.g., before surgery, before a standardized stress test). Thus, it remains unclear whether these findings can be generalized. Thus, research into the effects of music listening in various situations of daily life (in contrast to one artificial experimental situation) is warranted. To address this shortcoming, Ambulatory Assessment [3]—as a complementary tool to laboratory research—enables psychological phenomena to be studied in an ecologically valid way [4].

Ambulatory research investigating the stress-reducing effects of music listening in daily life is still in its infancy. Some of the available studies focused on the effects of music listening on emotion regulation in daily life [5–7]. In this regard, Juslin et al. [5] found that participants reported more positive emotions after having listened to music. The very few studies investigating associations between music listening and stress in daily life found that music listening does not reduce stress per se [8, 9]. Rather, situational factors may moderate this relationship: Music was only associated with stress reduction when other individuals were present while listening to music [9] or when music was specifically listened to for relaxation purposes [8]. Taken together, these studies illustrate the importance of capturing situational factors using Ambulatory Assessment to gain a deeper understanding of when and why music listening reduces stress.

However, as promising as these results might be, there are at least two shortcomings that deserve critical discussion: (1) Previous studies have relied on subjective music listening data only, and (2) there is a lack of studies investigating the temporal dynamics of the beneficial effects of music listening. Regarding the first shortcoming, most research using Ambulatory Assessment has been built around self-reports of music listening behavior, with participants being asked to indicate whether they had listened to music since the last data entry [e.g., 5, 10]. However, such data may be susceptible to retrospective memory biases [5]. Consequently, Juslin et al. [5] called for studies which objectively sample the music individuals listen to. To the best of our knowledge, to date only one study has objectively assessed participants’ music-listening behavior [6]. In this study, participants were prompted to answer questions regarding emotion regulation strategies and music listening whenever they started listening to music on their smartphone. The authors found that music listening was effectively used to regulate emotions. However, given the limited evidence of one single study, more research is clearly needed to test whether objectively assessed music listening behavior can reproduce the stress-reducing effects of music listening found in studies using self-report measures only.

The second shortcoming in the literature on music-induced stress reduction is that it is unclear how long participants must listen to music in order for its beneficial effects to unfold (duration of music listening) and how long it takes for music to exert stress-reducing effects (latency). This information is necessary in order to shed light on the temporal dynamics underlying the effects of music listening on stress. In experimental studies, the time intervals of music listening vary from study to study, spanning periods, for instance, from 10 min [11] to 45 min [12] and even more than 3 h [13]. However, there is no recommendation on how long music should be listened to in order to exert health-beneficial effects.

Concerning the latency of these beneficial effects in daily life, most previous studies used retrospective self-reports on music listening, and only a small number of studies simultaneously assessed either music listening and mood [7, 14] or current and past music listening [4, 15]. However, interpretations might vary depending on whether current or past music listening is reported. When current music listening is assessed, it is possible to make predictions about acute effects of music listening on stress (as both music listening and stress are assessed at the same moment). However, the assessment of past music listening captures time-delayed effects of music listening on stress, as music listening and stress are not assessed simultaneously. It would be intriguing to study whether the beneficial effect of music listening varies depending on whether acute effects (e.g., current music listening) or time-delayed effects (e.g., past music listening) are assessed. Moreover, regarding time-delayed effects, knowledge on the latency of the stress-reducing effects of music listening is completely lacking. Thus, the optimal time lag between music listening and the assessment of stress remains unexplored. For example, Linnemann et al. [8] reported that it was not possible in their study to determine how much time elapsed between music listening and the assessment of stress. However, to design specific music listening interventions for stress reduction purposes in daily life, research is needed to clarify the temporal dynamics underlying the associations between music listening and stress.

### The Present Study

Taken together, several problems remain unresolved in the literature on the relationship between music listening and stress. A critical methodological limitation inherent in most studies set in daily life is that they rely on (often retrospective) self-reports on music listening, which might be subject to memory bias. Therefore, an objective assessment of music listening behavior is warranted, which captures when individuals listen to music and for how long. This should enable researchers to investigate (a) which duration of music listening is necessary for stress reduction and (b) whether music listening has acute and/or time-delayed effects on stress. We therefore conducted an Ambulatory Assessment study to pursue the following two aims: First, we investigated whether there was still an association between self-reported stress levels and music listening when music listening was objectively tracked. Second, we explored the temporal dynamics underlying the association between music listening and stress in terms of duration (that is, the duration of music listening that is necessary to be associated with beneficial effects) and latency (that is, how long it takes for music to be associated with beneficial effects).

## Method

### Participants

We conducted a dual-site exploratory Ambulatory Assessment study with a total of *N* = 63 undergraduate students (study site 1: *n =* 37; study site 2: *n =* 26). Data from three participants (*n* = 30 single observations) were excluded because they completed fewer than 33% of the total signals [16]. Thus, the final sample consisted of 60 participants (37 women), aged 18 to 34 years (*M* = 22.4 years, SD = 3.5). Inclusion criteria were sufficient mastery of the German language and the ability to operate a smartphone. Participants were excluded if they indicated that they were pregnant, currently breastfeeding, or suffering from a mental disorder. The study protocol was approved by the local ethics committees at both study sites.

### Procedure

Informed consent was obtained from all individual participants included in the study. Basic demographic characteristics and self-reports on health using the Patient Health Questionnaire [17] were given from all participants. Then, participants received instructions on how to use the electronic diary device (iPod touch at study site 1; department- or participant-owned smartphones at study site 2). Since participants were instructed to listen to music only using the study device via the application “Simple Last.fm Scrobbler” (The SLS Team, 2016), the music files to which they intended to listen during the ensuing week were uploaded onto the electronic diary device. Then, the use of the application was explained. The “Simple Last.fm Scrobbler” application automatically logged the exact time point of music listening for any song that was listened to for at least half the duration of the track. The collected data on music listening for each participant were saved on the Last.fm servers (Last.fm Limited, London, UK). Starting from the next day, for a total of six (study site 2) or seven (study site 1) consecutive days, participants received six signals over a time window of 12 h, beginning at 10.00. Upon each signal, participants were asked to complete items concerning stress, mood, and music listening behavior, among others. Following recommendations of Hektner, Schmidt, and Csikszentmihalyi [18], this time window was divided into six blocks of 2 h, with the condition that consecutive signals were at least 30 min apart. If participants failed to respond to a signal or did not fully complete data entry, they were reminded twice with further signals. Participants were also able to postpone the signal if they were unable to complete the scheduled data entry at the current moment. Additionally, directly after waking up, participants completed a questionnaire on their electronic diary device, which included items on sleep quality, mood, and stress. However, data from this assessment are not included in the current analyses as no items on music listening were presented at this point. After completion of data collection, participants returned to the lab and were debriefed and reimbursed for their participation (either 20 euros or research credits).

A key advantage of Ambulatory Assessment methodology is its high external and ecological validity [19], as processes are investigated in their daily life [3]. The internal validity of this approach is limited, though, given the lack of rigorous experimental control, which renders causal inferences difficult. Ambulatory Assessment, thus, serves as a methodological approach that complements experimental studies. It opens up opportunities to assess ecologically valid data and allows for the identification of meaningful associative patterns.

Participants at study site 1 received an iPod touch with the pre-installed application “iDialog Pad” (G. Mutz, University of Cologne, Germany), which was used for presenting the items. The data were stored locally on the iPod touch and exported upon completion of the Ambulatory Assessment period when participants returned to the lab. Participants at study site 2 either received an Android-based smartphone with Android OS 5.0.1 (Google, Mountain View, CA, USA) or used their own Android-based smartphones. The data were collected via the movisensXS experience sampling application, version 0.8.4203 (movisens GmbH, Karlsruhe, Germany), which was downloaded and installed either on the personal or the department-owned smartphone at the beginning of the study. The data were stored locally and uploaded to a secure server when the smartphone was connected to the internet. The administration of the items was comparable when either using the iPod or the smartphone.

### Ambulatory Assessment Measures

#### Perceived Stress

To limit the burden on the participants, perceived stress (*M* = 1.17, SD = 1.02) was assessed using one item (“At this moment, I feel stressed,” 5-point scale ranging from 0 = not at all to 4 = very much; [20]).

#### Self-Reported Music Listening

For each signal, participants were asked whether they were currently listening to music (yes vs. no) and, if not, whether they had listened to music since the last signal (yes vs. no). Participants also indicated the duration of music listening on a 4-point scale: < 5 min, 5–20 min, 21–45 min, > 45 min.

#### Objectively Assessed Music Listening

The “Simple Last.fm Scrobbler” application collected information for each track (artist, title, date and time at the start of the track).

### Analytic Approach

To test for associations between self-reported music listening and stress, we created the dichotomous variable *self-reported music listening*, which was coded “1” if participants either indicated currently listening to music at the time of the signal or having listened to music since the last signal. It was coded “0” if they had not listened to music. To test for the latency with which self-reported music listening and stress might be correlated, we created another variable, *time lag*, which compared whether participants were currently listening to music (1) or had listened to music since the last signal (0) according to self-reports. This enabled an assessment of whether self-reported music was associated acute and/or delayed with stress.

In line with the variables on self-reported music listening, we created a binary variable, with current music listening or music listening since the last signal coded as “1” and no music listening in this time frame coded as “0,” respectively. Since Last.fm also stores exact dates and times, we generated the duration of music listening in this time frame (*M* = 36.7 min, SD = 33.9) and the time lag between the current signal and the most recent track (*M* = 46.5, SD = 44.6) as two continuous variables. This enabled us to quantify and directly test associations among the amount of music listening, the latency of music listening relative to the assessment of stress, and stress reports.

Since multiple data entries (Level 1) are nested within participants (Level 2), we computed two-level models with random intercepts in Stata 14 (Stata Corporation, College Station, TX, USA) and investigated within-person processes. Continuous independent variables on Level 1 were person-mean centered, and those on Level 2 were grand-mean centered [21]. We did not enter the averaged continuous Level-1 independent variables as a measure of between-subject processes, since we were primarily interested in within-subject processes only and thus wished to keep the model as parsimonious as possible [22]. Categorical variables were dummy-coded (reference of subjectively assessed duration: “< 5 min”).

We computed separate multilevel models for self-reported and objective music listening predictors to increase statistical power, since self-reported and objectively assessed data on music listening did not always converge. We first fitted the unconditional model, which revealed that 68.3% of the total variance in stress was attributable to within-person variability (ICC (type 1) = 0.317). Then, in a first step, we entered music listening as Level-1 predictor to test its association with stress. In the second step, we included the variables assessing duration of music listening and the time lag between the last track that was listened to and the current signal. Besides time, all analyses controlled for study site and gender given the outlined methodological idiosyncrasies between the study sites and the unequal distribution of gender across the study sites (*χ*^2^(1) = 2.37, *p* = .124, Cramérs V = .20). We additionally included the lag-1 serial autocorrelation, that is the stress level reported at the last signal.^1^ Finally, we did not include random effects of the Level-1 predictors, because their inclusion did not improve model fit (*χ*^2^(3) = 4.35, *p* = .226 for the model with the subjective data and *χ*^2^(3) = 2.76, *p* = .430 for the model with the objective data).

As there are no widely accepted recommendations for computing statistical power in multilevel models, we opted for the general recommendation of having at least 50 participants to obtain acceptable estimates of standard errors of predictors [23]. *P* values of < 0.050 were considered significant. All tests were two-tailed.

## Results

Compliance with the Ambulatory Assessment protocol was good [24], with participants completing approximately 39 signals on average (83.3% compliance). To test whether the compliance deteriorated over the course of the study, we computed a multilevel logistic regression, in which the outcome was whether a participant responded to a presented signal (“1”) or not (“0”). Day of study and the signal number of a given day were included as predictors. According to this model, compliance did not deteriorate over the day (OR = 0.96, *z* = − 1.52, *p* = .129), but did deteriorate over the course of the study (OR = 0.89, *z* = −2.86, *p* = .004).

### Descriptive Statistics

We compared the frequencies of self-reported and objectively assessed music listening episodes. Participants subjectively reported 692 episodes (38.5%) of music listening (either current or past music listening). However, the “Simple Last.fm Scrobbler” application registered only 486 episodes (26.9%) of music listening: Participants did not report 81 music listening episodes (4.6%) that were registered via Last.fm. At the same time, participants indicated 287 times (16.2%) that they were listening to music which was not registered via Last.fm. This indicates that participants possibly used other sources for music listening as well. Importantly, however, a chi-square test revealed a statistically significant relationship between self-reported and objectively assessed music listening, *χ*^2^(1) = 555.5, *p* < .001, Cramérs V = .56, which corresponds to a large effect size per convention (Cohen, 1988). The large effect size is also illustrated by an OR = 17.2, which indicates that it was 17.2 times more likely that an episode of music listening was registered via Last.fm. Thus, although there was a discrepancy between subjective and objective measures of music listening, the association between the two measures was significantly large and substantive.

### Self-Reported Vs. Objectively Tracked Music Listening

As indicated in Table 1, the first step of the multilevel logistic regression using the self-report data on music listening revealed a significant negative association between music listening and stress. Participants indicated lower levels of stress when they reported current or past music listening (*M* = 1.09, SE_M_ = 0.06) in comparison to no music listening (*M* = 1.19, SE_M_ = 0.06). However, objectively assessed music listening via the “Simple Last.fm Scrobbler” application was not significantly associated with stress. Participants indicated similar stress levels when they reported current or past music listening (*M* = 1.13, SE_M_ = 0.07) in comparison to no music listening (*M* = 1.10, SE_M_ = 0.06). Thus, although the subjective data replicated previous evidence that music listening is associated with stress reduction, this association was not replicated using the objective measure of music listening.

**Table 1:** Fixed Effects Estimates (Top) of Stress Levels as a Function of Music Listening, Temporal Distance and Duration

Fixed effects	Subjective Measures				Objective Measures			
	UC	*z*	*p*	*f*	UC	*z*	*p*	*f*
Step 1								
Music Listening	-0.10	-2.38	.017	.08	0.02	0.45	.652	.02
Step 2								
Duration	*χ*^2^(3) = 12.11		.007	.28	-0.003	-2.33	.017	.19
Time Lag	0.11	1.64	.101	.06	-0.002	-2.40	.017	.13

Stress was measured on a scale from 0 to 4. Duration represents how much participants listened to music between the current and the previous signal. Time Lag either compares music listening at the current signal with music listening prior to that (subjective measure) or the lag between the last played track and the current signal (objective measure). Chi-square tests reflect omnibus tests for the categorical subjective variable duration. All *p*-values are two-tailed

*UC,* unstandardized coefficient

### Temporal Dynamics of Stress-Reducing Effects of Music Listening

Both self-reported and objective measures of music listening duration were significantly associated with stress (Table 1). Participants reported significantly higher stress levels when they reported past music listening for less than 5 min (*M* = 1.15, SE_M_ = 0.09) or 5–20 min (*M* = 1.17, SE_M_ = 0.07) compared to music listening for 21–45 min (*M* = 0.98, SE_M_ = 0.09) or more than 45 min (*M* = 0.85, SE_M_ = 0.11). This was mirrored by the objective measure of duration, insofar as lower stress levels were associated with increasing duration of music listening. Compared to the subjective measures, the estimated marginal means showed that listening to music for less than 5 min was associated with the highest stress levels (*M* = 1.19, SE_M_ = 0.08) compared to 5–20 min (*M* = 1.16, SE_M_ = 0.08), 21–45 min (*M* = 1.11, SE_M_ = 0.07), and 60 min (*M* = 1.03, SE_M_ = 0.08). However, the effect size was larger for self-reported than for objectively assessed duration of music listening.

To test how long participants needed to listen to music in order to significantly detect an association with stress, we built another multilevel model with stress as the outcome and extended the categorical variable assessing duration by including data entries in which participants did not listen to music as the base category. This analysis is based on self-reported music listening. This model revealed that listening to music for less than 5 min, *b* = − 0.04, *z* = − 0.56, *p* = .577, and 5–20 min, *b* = − 0.04, *z* = − 0.07, *p* = .945, was not associated with reduced stress levels significantly, in contrast to listening for 21–45 min, *b* = − 0.18, *z* = − 2.44, *p* = .015, and more than 45 min, *b* = −0.34, *z* = −3.59, *p* < .001. Thus, these results suggest that music listening begins to be associated with reduced stress levels after around 20 min.

We also compared the descriptive statistics of the duration measures, which indicated that participants seemed to underestimate how long they listened to music. The mean objectively assessed duration of music listening was *M* = 24.5, SD = 19.5 when participants subjectively reported that they had listened to music for less than 5 min. Likewise, the objectively assessed mean duration was *M* = 33.2, SD = 28.3 when the self-reported duration was from 5 to 20 min, *M* = 46.9, SD = 36.84 for a self-reported duration of 21 to 45 min, and *M* = 81.1, SD = 50.2 for a self-reported duration of more than 45 min.

Next, we tested the latency of associations between stress and music listening. As a subjective measure of the latency of this association, we compared whether self-reported music listening at the current moment had a stronger association with reduced stress levels than having reported listening at a time point between the current and the previous signal. As indicated in Table 1, there was no significant difference for the self-report measure, insofar as stress levels did not differ depending on the time point of music listening. However, the objectively assessed time lag demonstrated a significant negative relationship with subjective stress levels, insofar as decreasing levels of self-reported stress were associated with an increasing time lag between the most recently played track and the current signal. These results demonstrate that beneficial associations between music listening and stress seem to occur in a time-delayed manner and not during the act of music listening per se.

## Discussion

The first aim of the present research was to investigate whether associations between music listening and stress in daily life can be found when using either self-reported or objectively assessed data on music listening. This was not the case: Whereas self-reports on mere music listening were associated with lower stress reports, objectively assessed data on mere music listening were not associated with reduced levels of stress.

Although the self-reported and objective measures of music listening were largely concordant, there were notable discrepancies. On the one hand, participants subjectively reported more music listening episodes than were objectively sampled, and on the other hand, in some cases, they did not report music listening subjectively even though the objectively assessed data indicated music listening. This discrepancy might explain why only the subjective measure of music listening was negatively associated with stress. There are some possible explanations for this discrepancy between subjectively and objectively assessed data on music listening. First, it might be that participants sometimes forgot to report music listening. Second, it is possible that music listening was objectively recorded but participants did not actively listen to it (i.e., participants did not stop the music when interrupted by another activity). Third, it could be the case that participants did not always use the smartphone application for music listening, for example at a concert. Another potential explanation for the different associative patterns of the subjective and objective assessment of music listening and stress might lie in previous findings that music-induced emotions are a prerequisite for music to exert beneficial effects [25]: When reporting music listening subjectively, participants may think about the music they listened to and thus engage with music on a cognitive and emotional level. For objectively assessed music listening, it is not clear whether the music was consciously listened to. This might explain why stress-reducing associations of music listening seem to be limited to music that is subjectively reported. Another explanation might be related to potential placebo effects of music listening as health-beneficial effects of music listening are frequently presented in the public discourse. Thus, in this regard, the association between music listening and lower subjective stress ratings might reflect shared method variance with participants relating music listening to beneficial effects in terms of positive music-induced emotions and lower subjective stress levels. Furthermore, as music is ubiquitous in daily life, it might not be possible to objectively assess all music listening that occurs in the soundscape of participants. Thus, the results do not provide clear evidence that subjectively reported data on music listening are better than objectively assessed data, as a certain proportion of music listening was probably not captured by the application. Instead, we believe that the results demonstrate that self-report measures of music listening are a reliable and valid method of registering music listening in daily life, as they capture a broader range of situations in which individuals listen to music and reflect more conscious acts of music listening. However, future research should explore new approaches to objectively assess music listening in additional applications, for example by using the Electronically Activated Recorder [26].

The second aim of the present study was to quantify how long individuals must listen to music in order to experience lower levels of stress and to explore the latency of these associations between music listening and stress. Concerning the duration of music listening, both subjectively and objectively assessed data revealed that with longer durations of music listening, individuals reported lower levels of stress, with a larger effect size for the subjective than for the objective assessment of duration. Our data further show that it seems necessary to listen to music for at least 20 min to show associations with stress. This association between duration and stress might explain why previous studies investigating the stress-reducing effects yielded inconsistent findings [1] as studies vary hugely in terms of the duration for which music was employed. Thus, future research should investigate the moderating influence of duration of music listening, e.g., by conducting a meta-analysis or by experimentally manipulating the duration of music listening.

Besides duration, another important factor of music listening in our study was the time lag between music listening and the assessment of stress. Whereas, on a subjective level, we only distinguished momentary music listening from past music listening; a more fine-grained analysis of the exact time lags between music listening and the assessment of stress revealed that stress levels decreased with increasing time lags between the most recently played track and the current assessment of stress. This finding does not indicate that the positive association between music listening and stress disappears over time, but rather indicates that it increases. It might be that other processes, events, and acts in the less-controlled context of daily life mask the acute short-term effects of music listening on stress found in laboratory research [e.g., 27]. Therefore, it might take some time before more persistent long-term associations between music listening and stress appear, similar to the results found for duration.

### Future Directions and Outlook

Findings from this exploratory study can lead to hitherto neglected methodological hypotheses on the associations between music listening and stress. Thus, these findings should inspire future studies on music listening and stress—both experimental and Ambulatory Assessment studies—in order to gain more empirical evidence on methodological factors that may determine associations between music listening and stress. Furthermore, in order to prevent shared method variance from influencing the pattern of results, future studies should measure stress from a multimodal perspective by means of psychobiological stress measures in addition to mere self-reports. In addition to these methodological issues, the identification of confounders and moderators (e.g., personality factors) and a more detailed analysis of situational factors (e.g., the experience of stress before listening to music) are necessary in order to gain insights into both between-subject and within-subject processes concerning the complex associations between music listening and stress in daily life.

### Strengths and Limitations

There are several limitations of the present study that warrant attention. First, as is common for intensive longitudinal data generated by Ambulatory Assessment, the correlational design cannot provide causal evidence for the effects of music listening on stress. Therefore, we cannot rule out alternative explanations, for instance that music listening co-varies with other factors possibly affecting stress. While our data cannot replace experimental studies on the stress-reducing effect of music listening, our study does provide evidence of the potential of such data for explaining the role of music listening on stress in naturalistic situations and confirms part of the experimental evidence outlined in the introduction with ecologically valid data. Second, to limit participant burden and to ensure compliance, we used short self-report scales, including a single-item measure of stress. Despite their frequent use in Ambulatory Assessment, single-item approaches limit reliability and predictive validity, especially of constructs that are multidimensional and broad in scope, such as stress [28]. Future research should assess stress more comprehensively. Furthermore, as stress is a multidimensional phenomenon, future studies should additionally assess physiological markers of stress to shed light on the mechanisms underlying the stress-reducing effect of music listening in daily life. Third, the variance of stress level was low, similar to findings from previous studies [9, 10]. Future studies should assess the associations between music listening and stress among different stressful situations, as was the case in a previous study in which students were investigated repeatedly during different stressful times of the university term [8]. Finally, the procedures at the two study sites differed in some aspects, such as smartphone or iPod touch application and study days, which may have affected the results. However, including study site as a control variable did not reveal significant differences between the study sites in the variables of interest, which, in our view, justifies the aggregation of the two samples.

### Conclusions

Our findings suggest that only subjectively assessed data on mere music listening was associated with lower subjective stress levels, possibly because the subjective reports captured more instances of music listening besides the smartphone/iPod application and because the participants re-engaged with the music on a cognitive and emotional level. However, when temporal dynamics of this association are of interest, subjectively reported data on music listening should be complemented by objective data on the exact time of music listening. Nevertheless, as music is ubiquitous in daily life, it remains challenging to objectively assess all music listening that occurs.

## Footnotes

1 Since participants reported lower levels of stress on weekends, we additionally included weekend as a binary control variable. However, this inclusion did not change the presented results, that is none of the presented significant results became insignificant and vice versa.
